# A Preoperative Virtual Reality App for Patients Scheduled for Cardiac Catheterization: Pre–Post Questionnaire Study Examining Feasibility, Usability, and Acceptability

**DOI:** 10.2196/29473

**Published:** 2022-02-22

**Authors:** Jiska J Aardoom, Alexander D Hilt, Tamar Woudenberg, Niels H Chavannes, Douwe E Atsma

**Affiliations:** 1 Department of Public Health and Primary Care Leiden University Medical Center Leiden Netherlands; 2 National eHealth Living Lab Leiden Netherlands; 3 Department of Cardiology Leiden University Medical Center Leiden Netherlands; 4 Department of Surgery Leiden University Medical Center Leiden Netherlands

**Keywords:** virtual reality, cardiac catheterization, stress inoculation training, preoperative anxiety, acceptability, feasibility, presence, immersive tendencies, presence, patient education, mobile phone

## Abstract

**Background:**

Pre- and postoperative anxiety is a common phenomenon associated with negative postoperative outcomes. Symptoms of posttraumatic stress disorder, such as fear, nightmares, and sleep deprivation, are prevalent in approximately 30% to 50% of patients following discharge from intensive care units after cardiac surgery. Preliminary evidence suggests a promising role of virtual reality (VR) in preventing stress-related reactions using stress inoculation training. Such training enables cognitive preparation of individuals for stressful situations, thereby becoming more tolerant and resistant to stress, subsequently reducing the risk of potential negative psychological consequences. This study investigated a preoperative VR app—*Pre-View*—aimed at better informing and preparing patients for cardiac catheterization.

**Objective:**

This study aims to assess the feasibility, usability, and acceptability of *Pre-View* in patients undergoing cardiac catheterization.

**Methods:**

Eligible participants were adults scheduled for elective cardiac catheterization. *Pre-View* comprised an interactive virtual representation of the whole care process related to cardiac catheterization, from entering the hospital for admission to postprocedural stay and discharge. These processes were represented through 360° videos and interactive photos. Self-report questionnaires were completed at baseline (ie, before catheterization and after undergoing the VR experience) and after cardiac catheterization. Outcome measures included user experience and satisfaction, VR presence and immersive tendencies, and user friendliness. The perceived effectiveness was assessed exploratively.

**Results:**

A total of 8 individuals, with a mean age of 67 (SD 7.5) years, participated in this study. Half of them underwent the VR experience at the hospital and the other half at home. Participants reported high levels of presence in the virtual environment (Presence Questionnaire score: mean 129.1, SD 13.4). The usability of *Pre-View* was well evaluated (System Usability Scale score: mean 89.1, SD 12.0), and patient satisfaction was high (Client Satisfaction Questionnaire score: mean 27.1, SD 3.2). Usability and satisfaction scores were higher for participants who underwent *Pre-View* at home versus those who underwent *Pre-View* at the hospital, although the latter group was significantly older; 72.8 versus 61.3, respectively. All participants reported *Pre-View* to be effective in terms of feeling better informed about the care process of cardiac catheterization. Most participants (7/8, 88%) reported *Pre-View* to be effective in terms of feeling better prepared for cardiac catheterization, acknowledging the potential of *Pre-View* in reducing negative psychological consequences after catheterization.

**Conclusions:**

The results provide initial support for the feasibility and acceptability of a preoperative VR app, creating a virtual environment that supports patient education and preparation for upcoming cardiac catheterization. More studies are needed to further investigate the effects of VR as a tool to better prepare patients for medical procedures, its effectiveness in reducing negative patient outcomes (eg, anxiety, stress, and postoperative recovery outcomes), and the generalizability of effects across different settings and patient populations.

## Introduction

### Background

Coronary artery disease is one of the 3 most common cardiovascular pathologies and plays a major role in mortality and morbidity worldwide [1]. The occlusion of coronary vessels can lead to myocardial infarction and eventually, death. Cardiac catheterization has evolved over many decades, drastically decreasing the number of deaths after acute myocardial infarction and relieving anginal complaints in an elective setting [2]. Overall, the clinical admission for such a procedure is short; however, psychological complaints regularly arise afterward. Approximately 30% to 50% of patients have been found to experience depression and symptoms of posttraumatic stress disorder (PTSD), such as fear, nightmares, and sleep deprivation, following cardiac surgery [3-6]. Such negative psychological outcomes can adversely affect patient recovery [3,7,8]. More specifically, studies have shown depression to be a strong risk factor for cardiac events, cardiac complications, and cardiac mortality following bypass surgery [3,9,10]. Furthermore, lower levels of quality of life and psychological functioning have been demonstrated in subgroups of patients reporting symptoms of PTSD after bypass surgery [5].

Previous research has demonstrated that preoperative education is a promising method to improve postsurgical outcomes, such as decreasing levels of anxiety and depression, improving recovery, and increasing patient satisfaction [11-15]. Preoperative patient education can be provided through verbal advice and written information. By informing and educating patients about the care process, such as surgery and hospital admission procedures, patients might feel more at ease and prepare for hospital admission and surgery accordingly.

The incorporation of multimedia tools has been suggested to be beneficial in terms of increasing patient satisfaction, perceived benefits, and understanding treatments [16-18]. New technologies such as virtual reality (VR) [19] is a successful tool in the education of patients [16-18]. Furthermore, VR can be used to desensitize patients to stressful events. VR exposure therapy is being increasingly used to treat PTSD and anxiety disorders [20-24]. Furthermore, preliminary evidence suggests that VR and stress inoculation training (SIT) can be successfully used to prevent stress-related reactions, such as PTSD. SIT can help prepare individuals for stressful situations (eg, as combat or battlefield stressors or medical emergencies or treatments) to reduce the risk of potential negative psychological consequences. When using VR during SIT, individuals can be pre-exposed to a stressor in a gradual and controlled manner. This is theorized to enable individuals to prepare themselves for an actual stressful event, thereby becoming more tolerant and resistant to stress. Indeed, using VR in the context of SIT, for example, has been shown to be a promising approach to prepare military personnel for combat situations [25-28], enhancing resilience, and potentially preventing PTSD-related symptoms.

In this study, a VR app—*Pre-View*—was used to investigate whether VR can be a useful medium in the preoperative management of cardiac patients undergoing elective cardiac catheterization. *Pre-View* combines preoperational education with virtual experience of the care process for elective cardiac catheterization in a Dutch university medical center. Using *Pre-View*, participants could virtually experience the whole process, from entering the hospital for admission until the moment of elective catheterization without showing the procedure itself, and to the postprocedural stay and discharge. The benefit of the VR experience over written or verbal information is that the patient is in control of the information he or she receives. The patient decides where to look and where to go to, and the app adjusts to that correspondingly. This increases the feeling of *being present* in the virtual environment, with *presence* referring to the subjective experience of being in a digital environment, while physically being in another [29]. The sense and quality of this presence are considered important factors for the efficacy of VR exposure therapy [30]. The quantification of presence can also be used as an evaluative measure for virtual experience [31].

### Objectives

This pilot study aims to assess the feasibility and acceptability of using the VR app, *Pre-View*, as a medium to inform and prepare patients for their upcoming elective cardiac catheterization.

## Methods

### Participant Recruitment and Eligibility Criteria

Participants were recruited from the Cardiology Department of the Leiden University Medical Center (LUMC), where they were listed for elective cardiac catheterization. Patients were eligible to participate if they were (1) aged ≥18 years; (2) able to speak and understand the Dutch language; (3) scheduled for elective cardiac catheterization; (4) able to undergo a VR experience, that is, not having impaired eyesight and a known history of epilepsy; and (5) not having undergone a previous cardiac catheterization. Participants were recruited and enrolled between January 6, 2020, and February 27, 2020.

### Ethical Considerations

The study was approved by the local medical ethics committee of the LUMC (protocol number: P19-068), and subsequently, a declaration of no objection was obtained from the Medical Ethics Review Committee. Interested participants received written information about the study and provided informed consent.

### Procedure

Potentially eligible patients (ie, aged ≥18 years and not having undergone previous cardiac catheterization) were approached and informed by email or telephone by a research intern (author TW). Subsequently, the VR experience was planned 1 or 2 weeks before the scheduled elective cardiac catheterization. Participants could choose to either undergo VR experience at home or at the hospital. In the hospital, participants were welcomed at the outpatient clinic for heart disease at the LUMC. When participants chose to undergo the experience at home, the research assistant visited the patient at home. Other than location, the process of undergoing the VR experience was identical. Patients were informed on how to use the VR app, after which they could independently undergo the experience. The research assistant was present to assist with any technical difficulties. Directly after completion of the VR experience, participants were asked to fill out a set of paper questionnaires assessing sociodemographic characteristics (ie, age and gender), presence, immersive tendencies, and questions related to satisfaction and usability (for more details, see the *Measures* section). Patients’ perceived effectiveness of *Pre-View* was assessed by a telephone call after cardiac catheterization to enable patients to reflect on whether and how *Pre-View* may have supported them during the process of preparation for the surgery, as well as during and after the catheterization.

### VR Experience: Pre-View

**Figure 1:**
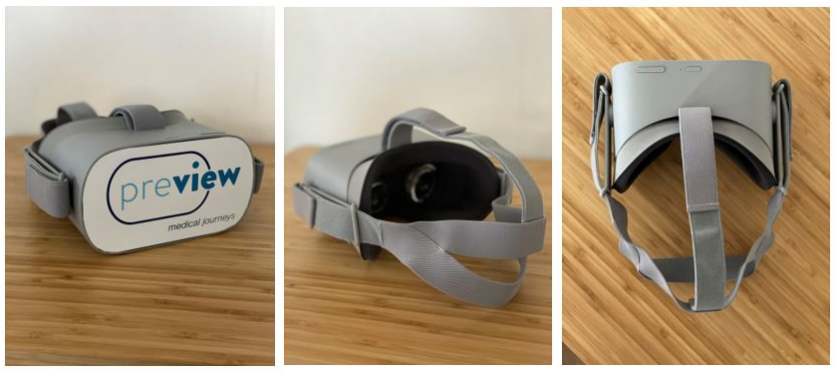
The Oculus Rift Go Virtual Reality headset was used in this study.

Patients underwent VR experience via a head-mounted display, the Oculus Rift Go device (Figure 1). The headset was individually adjustable even for participants wearing glasses. Within the VR environment, patients were provided with an interactive representation of the whole care process related to cardiac catheterization; in general, they could experience the day of heart catheterization. This encompassed the patient journey from entering the hospital for admission to the postprocedural stay. Heart catheterization itself was not presented but the related processes were as follows: patients were virtually transferred in a hospital bed with wheels to the operating room, where the cardiologist would briefly explain the procedure. The experience was represented through both video and interactive photos, which were captured and recorded during the development process of the VR app. Topics such as “What will happen in the ward?”, “What kind of clothing do I need?”, and “Who are allowed to stay?” as well as topics such as “What medication is given after the procedure?” and “Can I eat before the surgery?” were addressed during the experience. The experience was fully interactive; patients could choose objects or persons (eg, nurses or cardiologists) to gain more information on relevant topics on the care process at every stage of the stay. To do so, patients simply had to gaze for a few seconds at the object or person to select it. Hence, there was no need to press a button on the controller physically. For example, patients could gaze at the personnel around them when virtually lying in the hospital bed, after which an explanation would be given about the type of personnel they were (eg, nurse or cardiologist) and what their role during the stay or catheterization would be (to perform the procedure, to assist, etc). Further interaction took place through short quizzes, for example, choosing the right floor in the virtual elevator when patients need to find their way through the hospital toward the cardiology department. A detailed overview of the total experience is provided in Table 1. An example of a 360° photo can be found in Multimedia Appendix 1. All images and videos shown were context-specific, meaning that they were captured and recorded at the LUMC with actual LUMC staff to enhance feelings of relevance and realism. The VR experience lasted approximately 20 minutes, depending on the time a patient spent in each module.

**Table 1:** Overview of the virtual reality (VR) experience.

Virtual locations and procedures		Means	Description
**Part 1: hospital admission**			
	Hospital entrance	P^a^, I^b^, and A^c^	The hospital’s main entrance is shown, and the main menu and gaze function of the VR experience are explained.
	Route to elevators	P, I, and A	The hospital’s main hall is shown with the route to the hospital elevators being explained.
	Elevator entrance and ride	P, I, and A	The elevator entrance is shown and the choice of floor leading to the nursing ward is explained.
	Entrance of the cardiology ward	P, I, and A	The entrance of the cardiology ward is shown.
**Part 2: admission to cardiology ward and precatheterization procedure**			
	Cardiology ward counter	V^d^	The user virtually walks toward the counter of the cardiology ward, where the desk clerk welcomes them. The desk clerk asks for a hospital card and personal identification. Hereafter, the user walks toward the entrance of the patient room.
	Patient room: photo	P, A, and I	An interactive photo of the patient room is shown. Users need to collect items they will need to bring to the hospital (eg, clothing and phone-charger). After all items are found, the user is placed in a hospital bed.
	Patient room: videos	V	Two short videos are shown of a nurse and cardiologist, respectively, explaining the upcoming procedures.
	Transfer to operating room	V	The user is virtually being transferred in a hospital bed with wheels from the cardiology nursing ward to the operating room.
**Part 3: operating room**			
	Operating room	P, V, and I	A photo is shown of the interior of the operating room containing explanations of specific devices (eg, radiology equipment). After this exploration, the patient can start a video of the scrub nurse and attending interventional cardiologist. They explain the upcoming procedure in general, including what they will do during the procedure and what is to be expected of the procedure (eg, duration).
**Part 4: postcatheterization procedure at the cardiology nursing ward**			
	Patient room: inside hospital bed	V and P	A video is shown where the nurse and physician explain important aftercare issues and procedures. When the video is complete, the user can freely look around in the room and choose to be discharged when finished.
**Discharge**			
	Exit cardiology ward	V and P	A short exit video shows all personnel and wishes the patient the best of luck and a healthy recovery. Hereafter, the VR experience is finished, and the user is placed outside in front of the hospital.

^a^P: photo.

^b^I: interactive feature.

^c^A: audio.

^d^V: video.

### Measures

#### Presence

The participants’ degree of presence and immersion in the virtual environment was assessed using the Presence Questionnaire (PQ) [29]. The PQ quantifies the amount of focus that a person expends on objects or tasks generated by a digital app—in this case, the VR app. The PQ is the most commonly used questionnaire for measuring presence [32]. It was developed based on factors widely believed to underlie presence and was found to be highly reliable and internally consistent with the Immersive Tendencies Questionnaire (ITQ) [29]. It consists of 22 questions covering different elements on the level of presence, such as the degree of realism and immersion, the degree of involvement, how compelling the sense of mobility was inside the virtual environment, and the degree of control over the virtual environment. All questions were answered on a 7-point Likert scale, with answers ranging from *not at all* to *completely*. The total scores varied between 22 and 154.

#### Immersive Tendencies

Immersion refers to a state in which an individual experiences an environment as an integral part of it*,* thus being enveloped in it and interacting with it naturally. Immersive tendencies relate to the tendencies to become immersed or involved easily in virtual or *make-believe* situations, quantifying a person’s tendency to become immersed and focused in digital environments. In this study, the ITQ was used to quantify participants’ immersive tendencies [29]. The ITQ consists of 18 questions, mostly assessing the degree of involvement and focus in common activities (“Do you ever become so involved in a movie that you are not aware of things happening around you?” or “When playing sports, do you become so involved in the game that you lose track of time?”). Answers were rated on a 7-point Likert scale ranging from *never/not very well* to *always/very well.* Higher cumulative scores (range, total score 18 and 126) represented a higher immersive tendency to become immersed or involved easily in the virtual situation.

#### Satisfaction

Patient satisfaction was assessed using the Client Satisfaction Questionnaire-8 (CSQ-8) [33]. The CSQ-8 is a short, 8-item standardized global satisfaction measure, and each item can be scored on a scale of 1 to 4, with total scores ranging from 8 to 32. The mean satisfaction level was computed for each individual. The CSQ-8 is widely used in health care studies and has good reliability and validity [34,35].

Several questions were asked to further assess the satisfaction levels. First, participants were asked to rate their satisfaction with *Pre-View* on a scale of 1 (extremely dissatisfied) to 10 (extremely satisfied) and to briefly summarize and clarify their scores subsequently. Second, participants were asked to what extent *Pre-View* met their need in terms of received information on a 5-point Likert scale (1 *definitely not* to 5 *definitely*) and to briefly summarize and clarify their score subsequently. Third, participants were asked whether they experienced any discomfort or side effects when undergoing the VR experience (yes or no) and if yes, to elaborate on these.

#### Usability

To assess the usability of *Pre-View*, participants were asked to complete the System Usability Scale (SUS) [36]. The SUS provides a quick and reliable tool for measuring the usability of a wide variety of products and services. It comprises a 10-item questionnaire with 5-point Likert scales ranging from *strongly agree* to *strongly disagree*. The total SUS scores ranged from 0 to 100. The SUS is a reliable and robust tool for assessing usability [37].

#### Perceived Effectiveness

Participants were asked to what extent they agreed with several statements, assessing their perceived effectiveness of *Pre-View* regarding (1) feeling better informed about the care process of cardiac catheterization, (2) feeling better prepared for the care process of cardiac catheterization, and (3) reduction or prevention of potential negative psychological consequences (eg, nightmares, anxiety, and symptoms of depression) after cardiac catheterization. The answer scales ranged from 1 (*totally do not agree*) to 5 (*totally agree*).

### Statistical Analysis

All data were processed using SPSS (version 25). Descriptive analyses (ie, means, SDs, frequencies, and percentages) were used to describe the sociodemographic characteristics of the participants of the study population as well as to summarize the questionnaire data in terms of the measures described in the *Measures* section.

## Results

### Study Population

A total of 27 patients were approached to participate in this study, of whom 12 (44%) were interested in participating. Of the 27 patients, 1 (4%) patient dropped out of the study before undergoing the *Pre-View* because of fear of experiencing motion sickness. Furthermore, 11% (3/27) of patients were not included in the study because of cancelation of the VR appointment at the start of the COVID-19 pandemic and preventive measures that forced an early termination of study enrollment. This resulted in 30% (8/27) of patients who participated in this pilot study. Sociodemographic characteristics of each participant are shown in Table 2. The average age of the total study population was 67 (SD 7.5) years, including 75% (6/8) men and 25% (2/8) women. Half of the participants chose to undergo the VR experience at the hospital and the other half at home.

**Table 2:** Sociodemographic characteristics and outcome descriptives of individual participants.

Gender	Age (years)	Location	PQ^a^ score	ITQ^b^ score	CSQ-8^c^ score	SUS^d^ score
Male	73	Hospital	115.0	42.0	21.0	70.0
Male	77	Hospital	114.0	52.0	27.0	72.5
Female	73	Hospital	129.0	70.0	29.0	87.5
Male	68	Hospital	137.0	67.0	24.0	90.0
Male	60	Home	116.0	51.0	28.0	92.5
Male	69	Home	130.0	105.0	28.0	100.0
Female	59	Home	142.0	69.0	31.0	100.0
Male	57	Home	150.0	82.0	29.0	100.0

^a^PQ: Presence Questionnaire.

^b^ITQ: Immersive Tendencies Questionnaire.

^c^CSQ-8: Client Satisfaction Questionnaire-8.

^d^SUS: System Usability Scale.

### Presence and Immersive Tendencies

Patients reported high levels of presence in the virtual environment (Table 2), with an average PQ score of 129.1 (SD 13.4). At the individual item level, items that were scored lowest were related to how much one was able to control events (mean 4.3), the extent to which the visual display quality interfered or distracted one from performing assigned tasks or required activities (mean 4.6), and how much delay one experienced between their actions and expected outcomes (mean 4.9). Items that were scored highest related to how well one could concentrate on the assigned tasks or required activities rather than on the mechanisms used to perform those tasks or activities (mean 6.8), how involved one was in the virtual environment experience (mean 6.5), and how well one could actively survey or search the virtual environment (mean 6.5).

Regarding immersive tendencies, participants showed a mean score of 76.3 (SD 20.0), indicative of above average tendency of becoming immersed or involved in virtual or *make-believe* situations. Higher levels of immersive tendencies and presence were found in those who underwent VR experience at home (PQ mean 139.8; ITQ mean 80.8) than those who underwent VR experience at the hospital (PQ mean 118.5; ITQ mean 53.8); although, patients in the hospital group were, on average, approximately 10 years older than the home group; 73 years versus 61 years, respectively.

### Satisfaction and Usability

As shown in Table 2, the usability of the *Pre-View* app was well evaluated by all participants, with a mean SUS score of 89.1 (SD 12.0) on a scale of 0 to 100. Patient satisfaction as assessed by the CSQ-8 was high, with an average score of 27.1 (SD 3.2) on a scale of 8 to 32. The results of the additional assessments of participant satisfaction are shown in Table 3. These results demonstrated acceptable to good satisfaction with *Pre-View*. Positive remarks were mostly about the clear explanation and visualization of the procedure day. A patient elaborated on his score of 6 on the item “Overall, how satisfied are you with *Pre-View*?” (Table 3); he was not able to see all the videos during the experience because of a technical error resulting in a black screen. Also, there were 2 remarks on identifying targets for improvements. A patient had missed seeing the actual catheterization. Another patient indicated that the cardiologist in the VR experience could perhaps elaborate a little more about the diversity of complaints that one could raise, as only chest pain was stated as the reason for visiting the cardiologist. Finally, none of the participants reported side effects during or after the VR experience.

**Table 3:** Result in terms of satisfaction and perceived effectiveness of the virtual reality app.

Items		Answer scale	Values, n (%)	Values, mean (SD)
**Satisfaction items**				
	**Overall, how satisfied are you with *Pre-View*?**			8.6 (1.3)
		1=extremely dissatisfied	0 (0)	
		2	0 (0)	
		3	0 (0)	
		4	0 (0)	
		5	0 (0)	
		6	1 (13)	
		7	0 (0)	
		8	2 (25)	
		9	3 (38)	
		10=extremely satisfied	2 (25)	
	**To what extent did *Pre-View* fulfill your need in terms of information received before the cardiac catheterization?**			4.5 (0.5)
		1=not at all	0 (0)	
		2=not really	0 (0)	
		3=neutral or do not know	0 (0)	
		4=fairly well	4 (50)	
		5=really well	4 (50)	
	**Have any side effects occurred while undergoing *Pre-View* (nausea, dizziness, headache, etc)?**			2 (0)
		1=yes	0 (0)	
		2=no	8 (100)	
**Perceived effectiveness items**				
	***Pre-View* was effective in terms of feeling better informed about the cardiac catheterization care process**			4.5 (0.5)
		1=totally disagree	0 (0)	
		2=disagree	0 (0)	
		3=neutral or do not know	0 (0)	
		4=agree	4 (50)	
		5=totally agree	4 (50)	
	***Pre-View* was effective in terms of feeling better prepared for the care process of cardiac catheterization**			4.3 (0.7)
		1=totally disagree	0 (0)	
		2=disagree	0 (0)	
		3=neutral or do not know	1 (13)	
		4=agree	4 (50)	
		5=totally agree	3 (38)	
	***Pre-View* was effective, or could potentially be effective, in terms of reducing or preventing negative psychological consequences (eg, anxiety, nightmares, and symptoms of depression) after cardiac catheterization**			4.0 (0.9)
		1=totally disagree	0 (0)	
		2=disagree	0 (0)	
		3=neutral or do not know	1 (13)	
		4=agree	5 (63)	
		5=totally agree	2 (25)	

When looking at usability and satisfaction scores separately for the patients who underwent *Pre-View* at home versus those who underwent *Pre-View* at the hospital, both scores were higher for the former group: 98 versus 80 for usability scores and 29 versus 25 for acceptability scores. These subgroups differed, however, not only in terms of where they underwent the VR experience but also in terms of age; those who underwent *Pre-View* at the hospital showed a higher mean age (mean 72.8 years, SD 3.7 years) than those who underwent it at home (mean 61.3 years, SD 5.3 years).

### Perceived Effectiveness

As presented in Table 3, all patients agreed that *Pre-View* was effective in terms of feeling better informed about the care process of cardiac catheterization; half of the participants *totally agreed* with this statement, and the other half *agreed*. Furthermore, 7 (88%) of 8 patients agreed or totally agreed with the statement that *Pre-View* was effective in terms of feeling better prepared for the care process of cardiac catheterization. Overall, 25% (2/8) of patients who agreed elaborated: “If you know what is going to happen, you experience less stress” and “The more you know, the better.” Finally, when asked whether *Pre-View* has been effective or could potentially be effective in reducing or preventing negative psychological consequences after cardiac catheterization, of the 8 patients, 2 (25%) participants *totally agreed*, 5 (63%) participants *agreed*, and 1 (13%) participant *disagreed*. The patient who disagreed elaborated as follows: “Even though you are better prepared for what is going to happen, you still do not know exactly what they are doing during the procedure. Nor does it completely remove the anxiety about what they will find, which so there is always some uncertainty.” Another patient who agreed specifically remarked that even though he felt better informed and prepared, he still felt somewhat anxious and stressed before hospital admission for cardiac catheterization.

## Discussion

### Principal Findings

This pilot study investigated the feasibility, usability, and acceptability of a preoperative VR app (*Pre-View*) in the context of better informing and preparing patients for cardiac catheterization. Its feasibility was demonstrated by participants reporting high levels of presence in the virtual environment, and the VR experience was well tolerated without experiencing any side effects. Furthermore, the results indicate good user satisfaction and system usability. Finally, most participants self-reported *Pre-View* to have been effective in making them feel better informed, making them feel better prepared for the cardiac catheterization care process, and potentially reduce or prevent negative psychological consequences after cardiac catheterization.

The results of this study are promising in terms of feeling better informed about the hospital stay and corresponding elective cardiac catheterization. This is in line with previous literature suggesting that the incorporation of multimedia tools is beneficial for perceived benefits and understanding of upcoming treatments [16-18]. Our results also add to the body of literature underscoring the usefulness of VR as an engaging tool for patient education. For example, the results of a study by Pandrangi et al [38] showed that a VR experience modeling an abdominal aortic aneurysm for patients diagnosed with abdominal aortic aneurysm was perceived as beneficial in better understanding their health status and feeling more engaged in their health care. Another study demonstrated that patient education using VR training on radiotherapy increased knowledge and positive experiences of undergoing radiation therapy for patients with breast cancer [39]. Hence, the results of this pilot study underscore not only the acceptability and usability of using VR as a patient educational tool but also highlight the potential of using VR as a means to better inform patients about upcoming stressful treatment processes.

The preliminary results of this study suggest that *Pre-View* is potentially effective in preventing or reducing potential negative psychological consequences after surgery, which is compatible with the existing theory and body of literature indicating the potency of using VR technology as a means to desensitize individuals to stressful future events such as combat situations, thereby supporting resilience and preventing negative psychological symptoms [25-28]. Regarding hospital settings and surgery, few studies have investigated the effects of preoperative VR apps on patient outcomes. A single-blinded randomized controlled trial by Eijlers et al [40] investigated the effects of a child-friendly VR exposure to the operating theater on the day of children’s surgery, aiming to get them familiarized with the upcoming medical procedures (eg, anesthesia procedures and transfer to the operating room) and corresponding environment. VR exposure did not have a beneficial impact on anxiety levels during anesthesia and after surgery or on the levels of postoperative pain and emergence delirium. Nevertheless, a subgroup of children who underwent more painful surgeries (ie, adenoidectomy and tonsillectomy) were significantly less often in need of rescue analgesia when having received VR exposure than those who had not. Another randomized controlled trial investigated the effects of preoperative VR experience in patients undergoing cranial and spinal surgery [41]. In comparison to usual preoperative procedures, the VR experience was found to lead to higher patient satisfaction, better preparedness, and lower levels of stress on the day of surgery. Thus, based on the results of our pilot study and the limited available research discussed above, VR seems to be an acceptable and feasible preoperative preparation tool for use in hospital settings before medical procedures. However, further research is needed to establish its effects on both physical and psychological outcomes.

Future studies could further explore the effects of using preoperative VR experiences across different contexts (eg, type of medical procedures in different types of illnesses) and different patient demographics (eg, age, immersive tendencies, and psychological well-being status). In addition, the role of presence in patient satisfaction and outcomes may be an interesting direction for future research: Is presence a necessary precondition or moderator of patient satisfaction and outcomes in the context of preoperative VR interventions? Not feeling *present* in the virtual environment has been found to be associated with higher levels of dropout in VR treatment for anxiety disorders; however, the same review did not find an effect of the degree of presence on patient outcomes [30]. A final interesting direction for future research is to investigate whether preoperative VR interventions can be effectively delivered via smartphones. In the literature, the feasibility of smartphone-based delivery of VR has already been demonstrated for various goals. The *Cardboard* platform by Google has been used to deliver VR experiences successfully for educational purposes [42] as well as in the context of a smoking cessation program [43]. Google Cardboard is a foldout cardboard viewer that provides the structure for a head-mounted display, while the display is provided by a smartphone that can be placed inside the cardboard viewer. Such smartphone-based delivery of VR experiences is of interest because of its possibility for less costly and timely VR experiences, thereby enabling easier and broad-scale implementation as it would be more convenient for individuals to start and walk through the experience whenever and wherever they choose or prefer to.

### Limitations

The results of this pilot study should be interpreted in light of several limitations. The study sample size was small. Owing to the COVID-19 pandemic, appointments with included patients were canceled, and elective surgeries, including cardiac catheterization, at the time of study recruitment were postponed until further notice by the hospital. This led to premature termination of patient inclusion. The study was designed to assess the feasibility, usability, and acceptability of preoperative VR experience. Hence, no definitive statements can be made about the effectiveness of the VR experience in better informing and preparing patients for their upcoming hospital admission and corresponding procedures or in reducing negative psychological consequences afterward.

### Conclusions

The current results provide initial support for the feasibility and acceptability of a preoperative VR app, creating a virtual experience that can support patient education and prepare patients for upcoming coronary catheterization. Further studies are needed to investigate the effects of VR as a tool to better prepare patients for medical procedures, its effectiveness in terms of reducing negative patient outcomes after such procedures, and its effects across different settings and patient populations.

## Appendix

Multimedia Appendix 1 Several examples of (360°) photos of the virtual reality experience.

## Abbreviations

CSQ-8: Client Satisfaction Questionnaire-8
ITQ: Immersive Tendencies Questionnaire
LUMC: Leiden University Medical Center
PQ: Presence Questionnaire
PTSD: posttraumatic stress disorder
SIT: stress inoculation training
SUS: System Usability Scale
VR: virtual reality
