# Supplementary figures and images for: A Preoperative Virtual Reality App for Patients Scheduled for Cardiac Catheterization: Pre–Post Questionnaire Study Examining Feasibility, Usability, and Acceptability
Source: JMIR Cardio. 2022 Feb 22;6(1):e29473. doi: 10.2196/29473 (PMC8905473; doi:10.2196/29473)

**Multimedia Appendix 3**


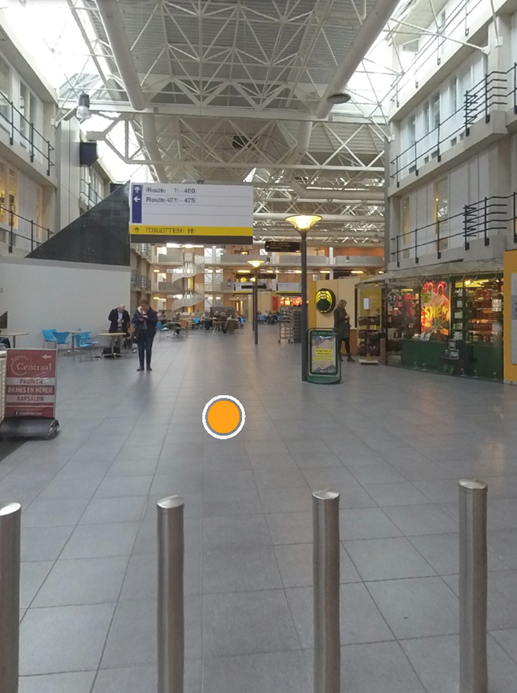


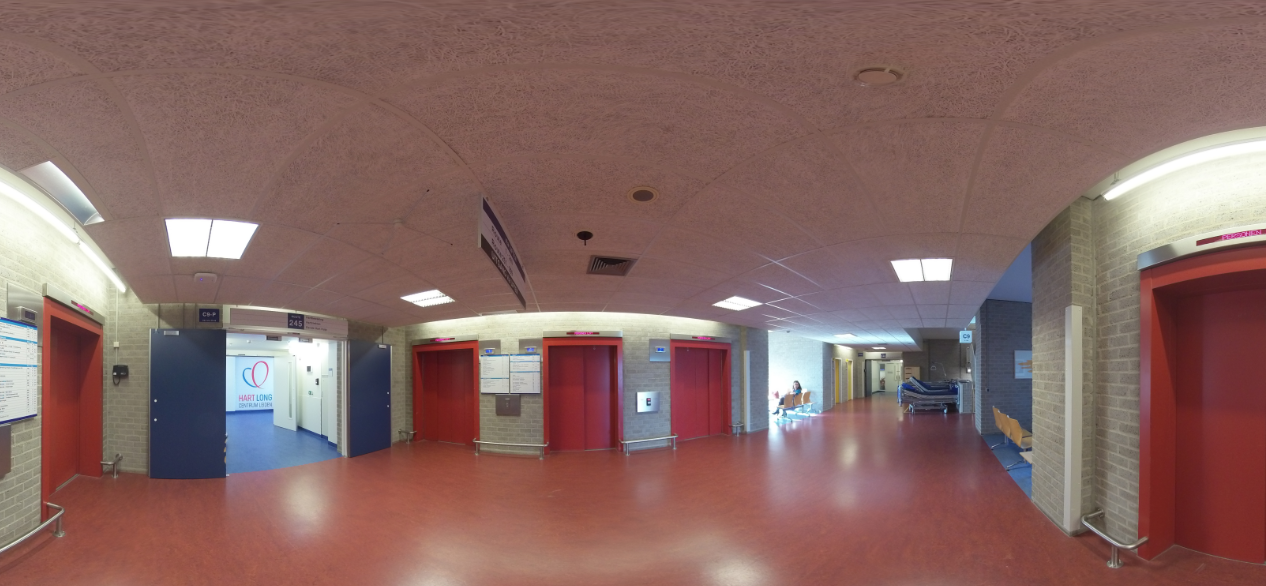


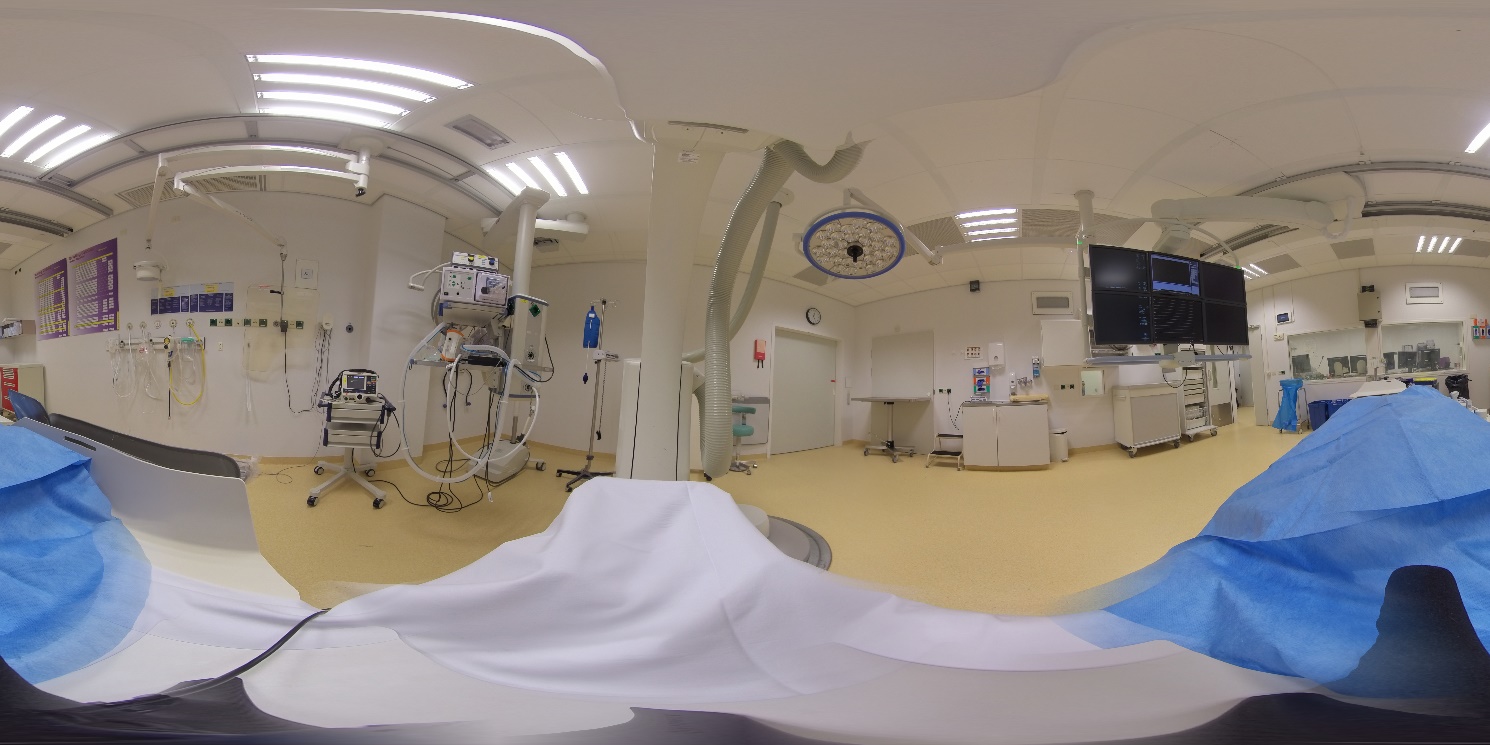

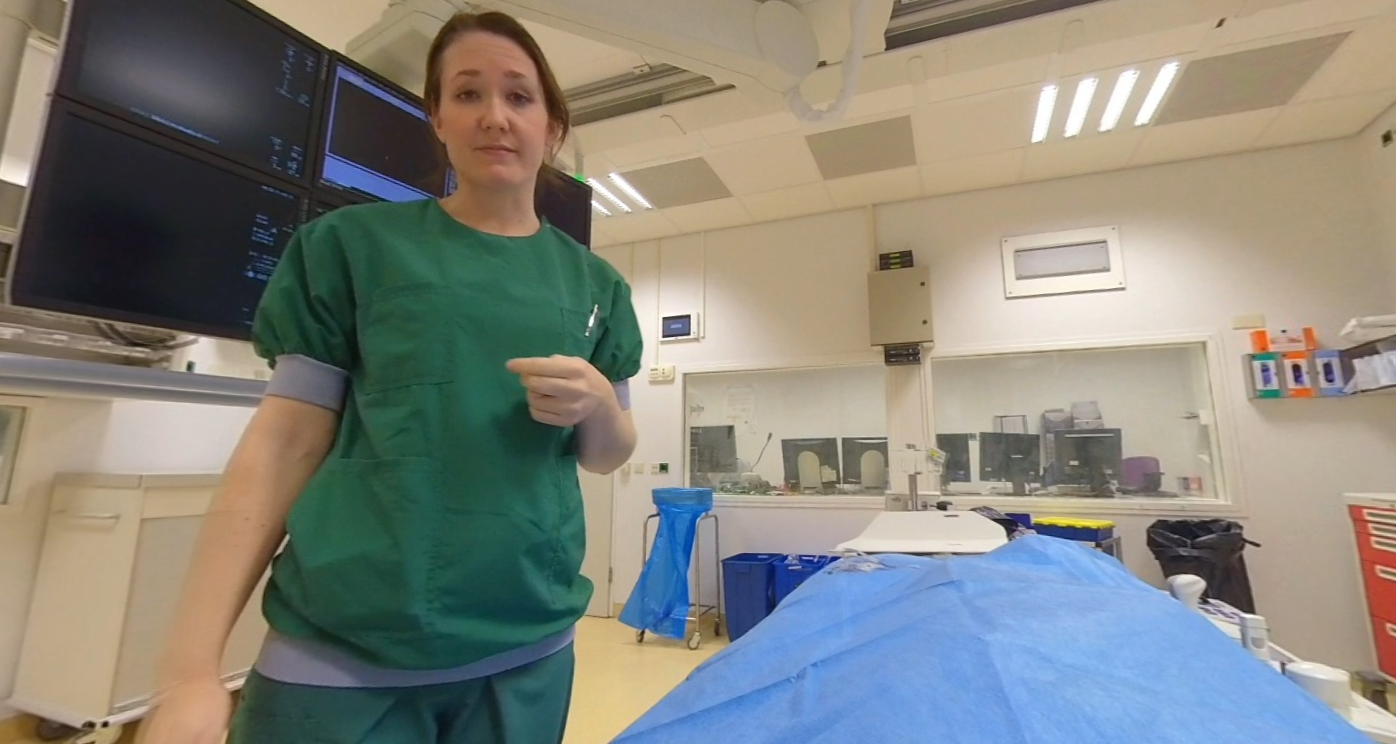

Supplement: Multimedia Appendix 1 [file cardio_v6i1e29473_app1.docx]
